# Self-organized criticality as a fundamental property of neural systems

**DOI:** 10.3389/fnsys.2014.00166

**Published:** 2014-09-23

**Authors:** Janina Hesse, Thilo Gross

**Affiliations:** ^1^Computational Neurophysiology Group, Institute for Theoretical Biology, Humboldt Universität zu BerlinBerlin, Germany; ^2^Bernstein Center for Computational Neuroscience BerlinBerlin, Germany; ^3^École Normale SupérieureParis, France; ^4^Department of Engineering Mathematics, Merchant Venturers School of Engineering, University of BristolBristol, UK

**Keywords:** self-organized criticality, brain, phase transition, dynamics, neural network

## Abstract

The neural criticality hypothesis states that the brain may be poised in a critical state at a boundary between different types of dynamics. Theoretical and experimental studies show that critical systems often exhibit optimal computational properties, suggesting the possibility that criticality has been evolutionarily selected as a useful trait for our nervous system. Evidence for criticality has been found in cell cultures, brain slices, and anesthetized animals. Yet, inconsistent results were reported for recordings in awake animals and humans, and current results point to open questions about the exact nature and mechanism of criticality, as well as its functional role. Therefore, the criticality hypothesis has remained a controversial proposition. Here, we provide an account of the mathematical and physical foundations of criticality. In the light of this conceptual framework, we then review and discuss recent experimental studies with the aim of identifying important next steps to be taken and connections to other fields that should be explored.

## 1. Introduction

The brain can be studied by two complementary approaches: Bottom-up approaches start on the level of single neurons or small groups of neurons, and then generalize upwards to the level of the brain. Hypotheses on the macroscopic level are formed based of the microscopic dynamics. For example, the observation of resonance in electrophysiological recordings can predict oscillations on the network level. By contrast, top-down approaches start by considering the properties of the brain on the level of brain areas or the whole brain, and infer downwards to the properties of its constituents. Hypotheses on the microscopic level are formed based of the macroscopic dynamics. For example, correlated activity in EEG recordings predicts a connection between the underlying brain areas.

A central concept connecting the microscopic and macroscopic levels is *criticality*. In the investigation of neural criticality, the word *critical* is used in the sense of statistical physics, which is distinct from other meanings, including the colloquial use. In statistical physics, criticality is defined as a specific type of behavior observed when a system undergoes a *phase transition*.

Physics characterizes the behavior of systems into qualitatively different *phases*. This classification scheme has its origin in the phases of classical matter, i.e., solid, liquid, and gaseous phase. The different macroscopic properties of, say, ice, liquid water, and steam can be explained by the microscopic forces between single water molecules. The discovery of this connection inspired the application of the concept of phases in a broader context and led to the identification of many more phases and different types of phase transitions.

**Figure 1:**
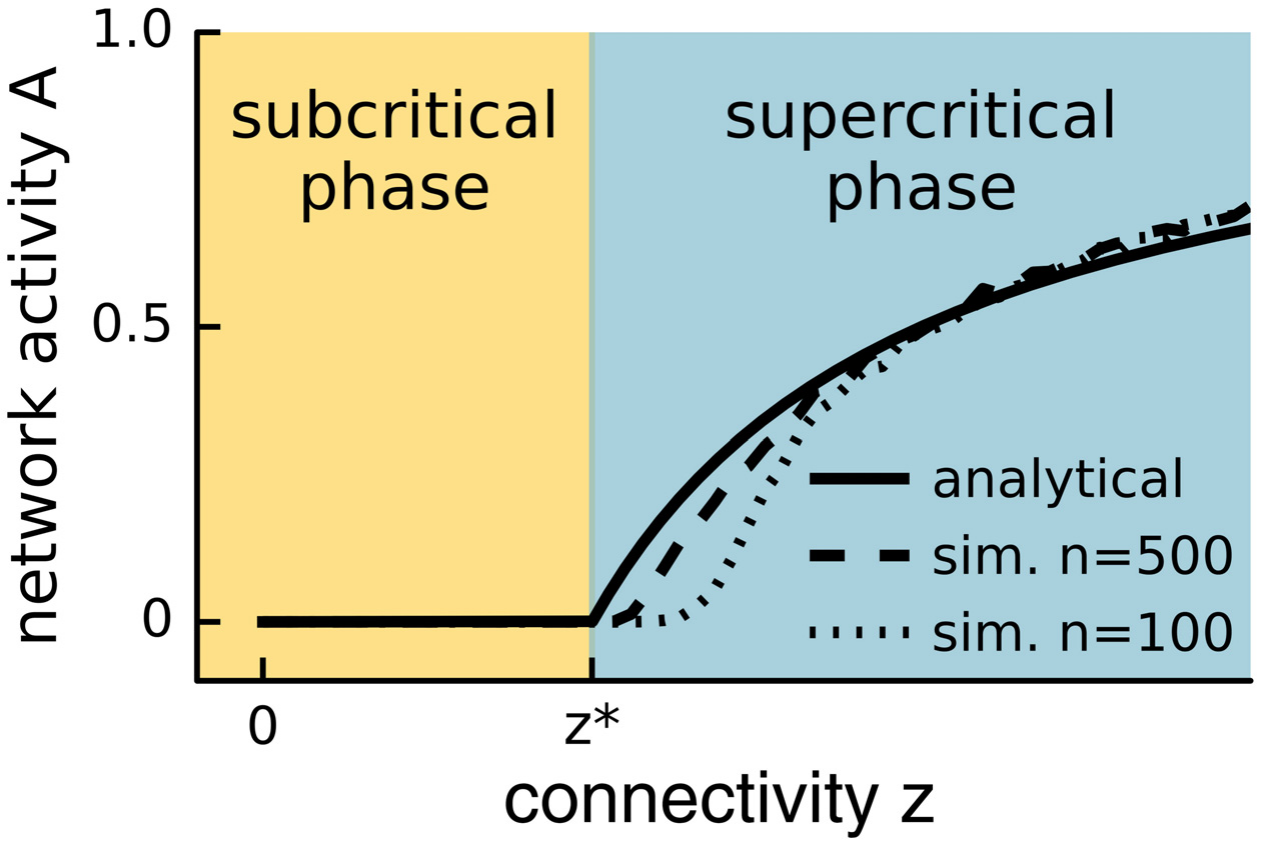
**Phase plot**. Network activity versus connectivity for the steady state solution of Equation (1) (straight line) and a simulated network with *n* = 500 (dashed line) or *n* = 100 (lower dashed line) neurons. A phase transition is observed at z^*^ (see main text) for the analytical solution with infinite *n*, whereas the transition appears in finite systems at slightly higher values of the control parameter and is smoothed out over a small interval. In the event-based simulation of Equation (1), the steady state network activity *A* was measured as *A* = *s*τ/*nT*, where *s* is the number of spikes recorded during the time period *T* following an initial relaxation period, and τ is the period over which the neuron remains active.

To distinguish different phases, one considers macroscopic, measurable properties of the system, so-called *order parameters*. One then observes how these order parameters change as an ambient property, the so-called *control parameter*, is varied. In general, a smooth change in the control parameter leads to a smooth change in the order parameters. However, there are certain points where the values of the order parameters jump or make sharp turns, see Figure 1. These points mark boundaries between different phases, and moving the control parameter across such a boundary causes a *phase transition*. If the transition is marked by a jump in the order parameters of the system (mathematically-speaking, a discontinuity in the phase diagram), the phase transition is called *discontinuous*. Such transitions are sometimes called *transitions of first order*. If the phase diagram is continuous and the transition is marked by a sharp corner (a point of non-differentiability), then the phase transition is *continuous* (*second order*).

If a system has a continuous phase transition, then the system can reside exactly at the transition point between two phases. This state on the edge between two qualitatively different types of behavior is called the *critical state*, and in this state the system is *at criticality*. Because phase transitions usually break certain symmetries of the system, they often separate an ordered state from a less ordered state. Critical states are therefore said to be on the *edge of chaos*.

As we discuss in detail below, systems at criticality are believed to have optimal memory and information processing capabilities. This general theoretical prediction was verified in many specific models such as boolean networks (Kauffman, 1984; Derrida and Pomeau, 1986), liquid state machines (Langton, 1990), and neuronal networks (Maass et al., 2002; Bertschinger and Natschläger, 2004), for a review also see Legenstein and Maass (2007). These findings inspired the *criticality hypothesis*, which proposes that the brain operates in a critical state because the associated optimal computational capabilities should be evolutionarily selected for.

Deviations from criticality could be symptomatic or causative for certain pathologies. This may pave the way for new diagnostics and treatments. For instance, Meisel et al. (2012) showed that hallmarks of criticality disappeared during epileptic seizures. Furthermore, insights into criticality in the brain could yield valuable design and operating principles for computation more in general, for example for unstructured artificial systems such as computers build from randomly-deposited nanowire memristors.

**Figure 2:**
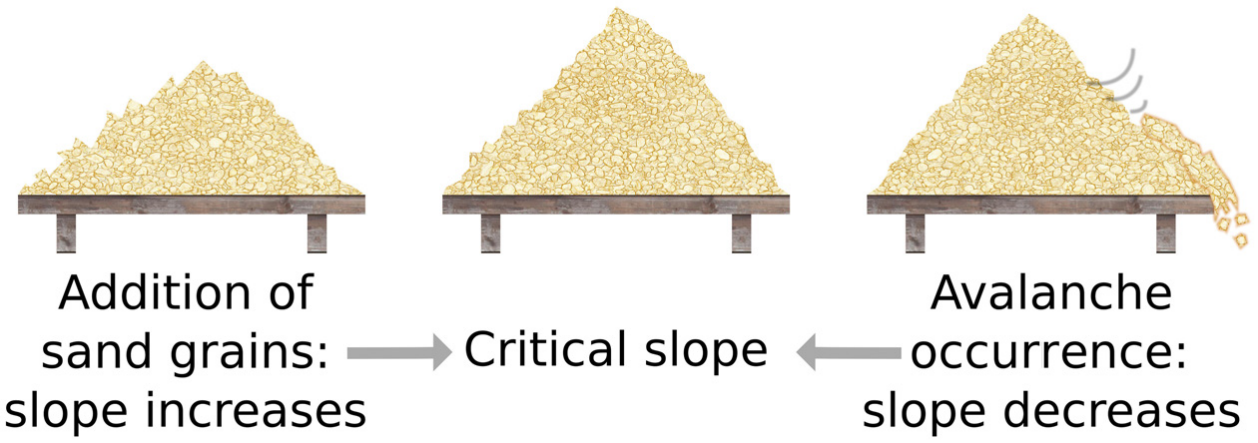
**The sandpile model**. The classical thought experiment motivating self-organized criticality is the sandpile model (Bak et al., 1988), which was experimentally reproduced using rice piles (Frette et al., 1996). Consider a pile of sand on a small table. Dropping an additional grain on the pile may set off avalanches that slide down the pile's slopes. The outcome of the avalanche dynamics then depends on the steepness of the slopes. Either all the sand will come to rest somewhere on the table or avalanches continue until some grains fall off the table's edge. In the former case, we have added one grain to the pile, so in average the steepness of slopes has increased. In the latter case, we have removed some grains from the pile, so in average the steepness of slopes has decreased. In the long run, the slopes evolve to a critical state where a single grain of sand that is dropped is likely to just settle on the pile, but also has a non-negligible probability to trigger a huge avalanche. This experiment already suggests that the critical state is very sensitive to stimuli, because a small (internal or external) variation can cause a large effect.

However, the criticality hypothesis is far from undisputed and many open questions remain. In particular, for a system to be at criticality, one parameter needs to be tuned exactly to the right point. One can therefore ask how a complex dynamic and variable system such as the brain can remain correctly tuned to this state. For a plausible answer, first note that the theory of phase transitions typically considers infinite systems. In large but finite systems, phase transitions occur not at a single point, but are smoothed out over a small parameter range. Instead of the unique critical state, we find a small region that is not technically critical, but still retains many properties of criticality, see Figure 1 (Moretti and Muñoz, 2013). However, even remaining in this “critical” region should require mechanisms that actively retune the brain. The general idea of systems tuning themselves to critical states through active decentralized processes is known as *self-organized criticality* (SOC) (Bak et al., 1988), and is illustrated in Figure 2. After a burst of activity in this area in the 1990s, the theory of self-organized criticality encountered some obstacles and interest slowly subsided (Vespignani and Zapperi, 1998). It was revived by Bornholdt and Rohlf (2000), who discovered an elegant mechanism of self-organized criticality in networks and already suggested it as a plausible mechanism for neural criticality.

The criticality hypothesis can thus build both on evolutionary arguments and on a plausible general mechanism that can explain the self-organization to the critical state. Although investigated analytically and numerically for numerous toy models, it is still unclear whether and how such a mechanism is implemented in the brain. Evidence for criticality has been found in experiments on cell cultures (e.g., Beggs and Plenz, 2003; Tetzlaff et al., 2010), animals (e.g., Petermann et al., 2009; Hahn et al., 2010) and humans (e.g., Kitzbichler et al., 2009; Meisel et al., 2012). However, it has been pointed out that some evidence may be misleading and could potentially be explained by alternative mechanisms (Botcharova et al., 2012). Some experimental studies also report negative results where characteristics of criticality were not observed in the neuronal activity (e.g., Bédard et al., 2006; Dehghani et al., 2012, but see criticism in Yu et al., 2014). In general, the relationship between the theoretical framework and its biological realization remains unclear. While models have demonstrated the plausibility of self-organized criticality in the brain, it is not clear to which of the many conceivable phase transitions the brain organizes, if and how different forms of plasticity drive the brain to this state, and whether different brain regions organize independently. Resolving these questions could lead to a much deeper understanding of neural criticality, explain apparent contradictions in experimental findings, and open up new connections with other fields.

Neural criticality has been reviewed in recent articles (Beggs, 2008; Kello et al., 2010; Beggs and Timme, 2012; Shew and Plenz, 2013; Marković and Gros, 2014) and is the topic of a contributed volume (Plenz and Niebur, 2014). In this review, our aim is to present a clear picture of the underlying concepts and ideas from statistical physics and nonlinear dynamics. We do not attempt to provide a comprehensive survey, but instead highlight specific papers to illustrate general insights that are evident in much of the recent literature. We first present a simple toy model that provides the essential concepts in front of which much of the recent work can be discussed. We then review self-organized criticality in nervous systems with a special focus on the interaction of theoretical and experimental work in this field. We point out several current questions and connections to other phenomena. Because of the emerging connections, we believe that the criticality hypothesis inspires discussions and the development of tools for the analysis of brain dynamics which will proof useful independent of the validity of the hypothesis itself.

## 2. Example of a phase transition in a network

Phase transitions and criticality can already be observed in simple network models. In physics, such highly simplified models have proven useful to distill the essence of a phenomenon, before investigating how this essence is reshaped through additional details present in the real system.

Consider a large directed network of excitable nodes that can be seen as a crude model of neurons. In average, each node has *z* outgoing links that can propagate activity to other nodes. In analogy to neural systems, we refer to the source of a given link as the *presynaptic* node and to the destination of a given link as the *postsynaptic* node. For a given link, activity is not transmitted instantaneously; instead, there is a small probability *p* that an activation of the presynaptic node activates the postsynaptic node in a small time interval of length τ. Active nodes decay back to the inactive state within the same time interval.

Clearly, this model is excessively simplistic and omits many additional effects and factors that are present in real nervous systems. However, as we show below, the model already contains all ingredients to exhibit a phase transition, and thus provides us with a simple model of a phase transition to play with.

Let us now try to understand the macroscopic dynamics of the system based on its microscopic rules. We define the network activity *A(t)* as the mean proportion of activated nodes at time *t*. Higher values of *A* imply that there are more active nodes, which can serve as sources of activity, but less resting nodes, which can still be activated. Mathematically, we can capture the ensuing dynamics by the differential equation

(1) $$\frac{dA}{dt}=\underset{\text{inactivation}}{\underset{︸}{-\;\frac{1}{\tau }A}}\underset{\text{activation}}{\underset{︸}{+\;pz\left(1-A\right)\frac{1}{\tau }A.}}$$

The system approaches a dynamical equilibrium $\frac{dA}{dt}=0$. Setting the left hand side of Equation (1) to zero reveals two qualitatively different steady states. In one of them, the activity dies out, *A*_0_ = 0, whereas in the other, a stable level of activity *A*_0_ = 1 − 1/*pz* is maintained. Generally, a system will only approach steady states which are stable to small perturbations. Stability analysis (Guckenheimer and Holmes, 1983) reveals that the stable state is the quiescent state for *pz* < 1 and the active state for *pz* > 1, such that the activity is non-negative. We can thus say that we observe an active state of the network when the connectivity *z* is greater or equal than z^*^ = 1/*p*.

Plotting the level of activity observed in the system's long term behavior, *A*_0_, as a function of the connectivity *z* reveals a typical *phase diagram* (Figure 1). In this context, the connectivity *z* is the control parameter, and the activity *A*_0_ is the order parameter. The diagram shows a subcritical quiescent phase and a supercritical active phase. The critical connectivity z^*^ = 1/*p* corresponds to a phase transition between these two phases. We note that even in this simple model the relation between phase transitions and symmetry breaking is evident. In the quiescent phase, all nodes are in the same (inactive) state, whereas this symmetry is broken in the active phase. In the quiescent phase, the system is completely static, whereas in the active phase, the individual nodes are activated stochastically, and seemingly chaotically. The phase transition point therefore marks the edge of chaos.

The analytical solution only holds for the limit of large networks. In small networks, network activity is difficult to sustain near the critical point, where the sustained network activity is so low that it easily dies out by chance. The abrupt change at the critical point is smoothed out and the observed phases are no longer perfectly distinct (Figure 1). Analog effects are seen in any finite system. In a large but finite system such as the brain, one would therefore not expect to find a single isolated point that expresses perfect criticality, but rather a small region that shows properties of critical systems in an approximate sense.

We emphasize that the simple model discussed here only exhibits one type of phase transition, the onset of activity. Additionally, there can be many other types of phase transitions. Another example that is commonly encountered in models, and may be more relevant for neural information processing, is a transition that marks the onset of synchronous (i.e., correlated) activity in the network (e.g., Meisel and Gross, 2009; Yang et al., 2012). One implication of the presence of such additional transitions is that labels such as subcritical and supercritical can only be applied with respect to a certain transition. For instance, a system that shows activity, but not correlated activity, can be considered supercritical with respect to the activity transition, but subcritical with respect to the synchronization transition.

## 3. Properties of phase transitions

In the following, we discuss how phase transitions and critical dynamics can be detected in experiments. The most direct evidence for a phase transition is certainly provided by a phase diagram (Figure 1) (Dickman et al., 2000). In this type of diagram, the existence of a phase transition can be seen directly in the response of the order parameter to variations of the control parameter. However, for creating such a diagram the control parameter must be accessible (controllable) in the experimental setting. For instance, it is difficult to imagine an experiment where the connectivity of the brain (our parameter *z* from above) can be varied *in vivo*. Yet, it might be possible to control the effective connectivity (e.g., *pz*) by pharmacological interventions in *in vitro* experiments. Although some studies discussed below report results for such modifications of control parameters, most of the evidence for criticality comes from experiments that show criticality indirectly by the observation of certain hallmarks. In this section, we discuss these hallmarks of criticality in the context of the simple model introduced above.

One commonly used hallmark comes from the theory of branching processes (Harris, 1963). Suppose we could observe only a tiny portion of the system, which only rarely lights up with (possibly spontaneous) activity. Under the assumption that the connections are sufficiently short-ranged to be within our observation window, we can still estimate the number of secondary activations that a given focal activation triggers, the so-called *branching parameter* σ. In the subcritical phase, this number is in average less than one. In the supercritical phase, the dynamics persists in the system and thus there must be in average as many activations as deactivations, which implies a branching parameter of one. In a spatially extended system that is not too far in the supercritical phase, a branching parameter greater than one may be observed over short times in response to an artificial excitation. Therefore, the observation of a branching parameter σ = 1 in response to an artificial excitation in a sufficiently quiescent system may be seen as evidence for criticality. However, compared to other hallmarks, this evidence is relatively weak because a branching ratio of one does not necessarily imply critical dynamics, but is also observed in supercritical states.

Another hallmark of criticality is related to the response of the system to external stimuli. In our model, the sensitivity to inputs (the *dynamic range*) is maximal at criticality. This can be shown by considering the temporal development of a small perturbation δ. The dynamical evolution of the perturbation of the steady state *A*_0_ is given by inserting *A*_0_ + δ into Equation (1), which yields

$$\begin{array}{l} \frac{d\delta }{dt}=\left(\;-1+pz\left(1-(A_{0}+\delta )\right)\;\right)\frac{1}{\tau }(A_{0}+\delta )\\ \;=-\frac{1}{\tau }\left(\;\pm (1-pz)\delta +pz\delta^{2}\;\right)\\ \;\approx -\frac{1}{\tau }\left(\;\pm (1-pz)\delta \;\right), \end{array}$$

where the plus applies if *z* < 1/*p* and the minus otherwise. The approximation in the last line holds for sufficiently small perturbations. The resulting equation is a linear differential equation, which implies that after the perturbation the system relaxes rapidly (exponentially) back to *A*_0_. In this case, the half-life of a sufficiently small perturbation is |τ ln(2)/(1 − *pz*)|. Any memory of the perturbation disappears therefore quickly. When the system approaches criticality, *pz* → 1, such that the half-life increases. At criticality, z^*^ = 1/*p*, such that the first order term 1 − *pz* vanishes, and the approximation leading to the third line no longer holds. In this case, the system relaxes only geometrically back to the state *A*_0_, which means that the memory of the perturbation is retained for a long time. This property is often called *critical slowing down*. Let us emphasize that critical slowing down is not only a property of the specific model considered here, but a general feature of critical phase transitions in the dynamics of a system (Scheffer et al., 2009). It lends critical systems a long memory and may play an important role for their computational properties.

In the example system, we can also understand the emergence of memory in the critical state on a microscopic level. Consider a situation in which we artificially activate a small number of neurons. We now ask how long the memory of this activation lasts in the time evolution of the system. Let us first consider a system in a subcritical state. Here, we already know that the branching parameter is less than one and hence the initially activated neurons will activate only a smaller number of neurons such that the signal from the initial activation quickly (i.e., exponentially) decreases over time. Consider now a supercritical state. We recognize that the branching parameter is equal to one, so we expect that the initial artificial activation of the small group of neurons triggers a cascade that stays in average roughly constant in size. We could therefore naively expect that the memory of the activation persists in the system. However, the truth is a bit more subtle: While the cascade indeed persists, some of the neurons involved in the cascade would have been activated anyway due to the ongoing self-sustained activity of the system. Thus, the difference between the artificially excited system and a system where the artificial activation did not take place shrinks in time; again the memory of the activation is lost exponentially. By contrast, the critical system already has a branching parameter of one, allowing the cascade that we have set off to persist for a long time, and it has also negligible background activity, allowing the information transmitted by the cascade to persist without interference.

The slowing down leads to another observable characteristic of critical systems, called 1/*f*-noise, which is commonly observed in nature (Hausdorff and Peng, 1996). If a critical system is constantly perturbed by weak random inputs, the dynamics is a superposition of a multitude of geometric responses. The power spectrum of this noisy response then follows a power-law, which means that the energy dissipated at frequency *f* is approximately 1/*f*^α^, where α is some constant. While every critical system should exhibit power-law noise, the observation of this type of noise alone does not constitute a proof of criticality, as it is also observed in certain other processes (Cencini et al., 2000; Bédard et al., 2006).

Power-laws appear in critical systems also in a different way. Loosely speaking, phase transitions occur at the points where the line between macroscopic and microscopic dynamics is blurred, e.g., where avalanches initiated on the microscopic level become so large that they affect the dynamics on the macroscopic level. For several reasons this is only possible when the size distribution of avalanches obeys a power-law (Levy and Solomon, 1996). Let us once again consider the simple model proposed above. Since the branching ratio is one independent of the size of the current avalanche, the probability distributions describing the cascades of events downstream from an activated node are independent of whether the node is the initial node that sparked the avalanche or a node that is only activated as the result of a long sequence of events. This is one aspect of the self-similarity found in critical processes (Marković and Gros, 2014). The cascade of subsequent activations caused by a given node is statistically identical to the cascades of subsequent activations triggered by the activated nodes. This in turn causes power-laws to appear in many observables of the system. Thus, criticality is generally associated with the appearance of power-law distributions of the form *f(x)* = *Cx*^−α^ for many different observables.

The observation of power-laws in multiple observables with consistent exponents constitutes a relatively strong proof for criticality. Although it is often pointed out that also these relationships could arise in non-critical systems, this criticism is much weaker for microscopic observables than for macroscopically recorded power-law noise. We note that some of the examples that are often quoted for spurious are wrong. For instance, it is often held that the Barabasi-Albert model (Barabási and Albert, 1999) leads to networks with a power-law degree distribution but does not correspond to a critical state. However, the Barabasi-Albert model is indeed in a critical state that marks the transition between exponential and star-like networks (Krapivsky and Krioukov, 2008).

A consequence of the blurring of the line between global and local scales, and part of the reason for the appearance of power-laws, is the so-called *scale independence* (Goldenfeld, 1992). This phenomenon captures the observation that critical systems show similar patterns at all scales. For example, the shapes of avalanches of any size resemble each other (Marković and Gros, 2014). As one approaches criticality, correlations occur between distant parts of the system, which means that external perturbations or spontaneous fluctuations can influence large parts of the system. For instance, stimulations induce small avalanches already in the subcritical region of our simple model. As we slowly increase the connectivity, these avalanches get bigger and bigger and reach the scale of the system at criticality. In this case, the avalanches occur on all scales up to the system size, which implies that the typical length of correlations diverges. If we increase the connectivity further, activity in the system continues to increase, making simultaneous occurring avalanches likely. As a node cannot be activated twice at the same time, one of the avalanches effectively stops whenever two avalanches reach the same node. These collisions between the avalanches decrease long-ranged correlations and destroy the divergence of the correlation length.

In summary, criticality occurs at phase transitions for which the order parameter changes non-smoothly but continuously with the control parameter. Proper phase transitions are an idealization only expected in the infinite size limit—in real systems, the transition is less well defined and smoothed out over a finite interval. At criticality, as well as in its proximity, the system dynamics exhibits critical slowing down, and the distributions of observables and fluctuations follow power-laws. These hallmarks of criticality lend critical systems their optimal information processing and storage capabilities, reviewed by Shew and Plenz (2013). Critical slowing down allows memories of dynamical patterns to be retained for a long time (Beggs and Plenz, 2004; Haldeman and Beggs, 2005; Chialvo, 2006; Chen et al., 2010; Kello et al., 2010). Furthermore, criticality maximizes the dynamic range of the response to inputs (Kinouchi and Copelli, 2006; Shew et al., 2009) and the variability of the neuronal response (Shew et al., 2011; Yang et al., 2012; Meisel et al., 2013). As scale-independent systems naturally show both small and large activity patterns, inputs can be processed in parallel, and integrated over the whole system (Gutiérrez et al., 2011).

## 4. Self-organization to a critical brain state

To observe criticality, a control parameter has to be tuned to its critical value. In a variable system such as the brain, and without an external observer, critical dynamics can only be conserved by *self-organized criticality* (Bak, 1996), a constant tuning of the control parameter by a decentralized internal mechanism. For many systems with a critical phase transition, *self-organized criticality* is easily implemented by a mechanism that increases the control parameter in the subcritical phase and decreases it in the supercritical phase.

We use the term *control parameter* also in self-organized critical systems although the control parameter is no longer controlled externally, but by the system itself. To adjust the control parameter appropriately, self-organizing mechanisms have to evaluate the current phase of the system from an internal perspective. In the nervous system, the self-organization probably relies on the dynamics of single neurons or synapses, and not on a global regulation, e.g., by the endocrine system, because evidence for critical brain dynamics is especially prominent in *in vitro* studies, where the neurons are separated from the rest of the brain that could act as global integrator. A central challenge is therefore to explain how individual neurons or synapses can infer the phase from local observations.

To decide whether the system is in the sub- or supercritical phase, the self-organizing mechanism has to evaluate the global mean of the order parameter. However, as the information accessible to a single neuron or synapse is necessarily local, it is reasonable to expect that the global mean is approximated by a temporal mean over the dynamics (Bornholdt and Rohlf, 2000). To allow for an estimation of the global mean based on a temporal integration of local observations, the change in the control parameter has to be considerably slower than the dynamics of the system. For example, in the sandpile model presented in the introduction (Bak et al., 1988), criticality is only reached when the next sand grain is dropped after any dynamics on the pile has ceased. Self-organized critical systems show in general a *time-scale separation* between changes in the system structure and changes in the dynamics of the system (Vespignani and Zapperi, 1998).

Theoretical arguments seem to suggest that self-organized criticality can be fully realized only in systems in which the control parameter is conserved (Dickman et al., 2000). In the brain, which is constantly subject to external input, the self-organization never precisely reaches the critical point (Bonachela et al., 2010). However, the characteristics of criticality, such as computational capabilities and sensitivity, are already increased in the proximity of the critical point. Therefore, we use the term self-organized criticality to refer both to neural networks which are right at or sufficiently close the critical point, a state that has previously been called *self-organized quasi-criticality* (Bonachela and Muñoz, 2009).

For the brain with its highly hierarchical and modular structure, it is likely that critical points generalize to critical regions (Griffiths phases) (Moretti and Muñoz, 2013). This relaxes the requirements on the tuning of the control parameter, which could also be shown in a realistic model of neuronal network dynamics (Rubinov et al., 2011). In modular systems, the global phase transition is spread out because, for a certain range of the control parameter, some modules are still in the subcritical phase, while other modules are already in the supercritical phase. Properties arising at criticality, such as power-law distributions, large dynamic range and slowing down of the dynamics, are approximately observed for any value of the control parameter in this critical range. Dynamical states similar to criticality are therefore likely whenever the self-organizing mechanism tunes the control parameter to the proximity of this critical region.

Self-organization of plausible neural models to criticality was demonstrated in a number of papers (e.g., Bornholdt and Rohlf, 2000; Levina et al., 2009; Meisel and Gross, 2009; Droste et al., 2013). However, many questions remain. First, we do not know which parameters in the brain are tuned to reach criticality. On a microscopic scale, synaptic conductances seem to be a likely candidate. As a substantial change in the synaptic conductances is only observed after several spikes, the plasticity acts on a slower time-scale than the neuronal activity. This provides the time-scale separation required for a robust tuning of the system to critical states. A change in the synaptic conductances could directly influence the excitability of the synapse (basically the parameter *p* in our simplified model), which is sufficient to tune the system to criticality.

A change of individual synaptic weights, which translates into an overall change in excitability, is only the simplest possible scenario. The excitability can also be changed directly by mechanisms of homeostatic plasticity (Stewart and Plenz, 2008; Droste et al., 2013). Other possible targets for sophisticated self-organizing plasticity mechanisms are changes in the level of micro-scale modularity, or of the heterogeneity in the system. These factors, which we have ignored so far, affect the location of critical points and can thus be used to tune the system to criticality. The simple picture, in which exactly one global control parameter is tuned, is thus misleading. In reality, the microscopic changes in the system are likely to affect tens or hundreds of network level quantities at the same time, which all act as possible control parameters for phase transitions.

Another open question is to which critical state the network organizes. While we have so far focused on the phase transition at the onset of activity, some evidence suggests the onset of synchrony as a more likely candidate. Some insights into this question can be gained based on the relation between the nature of the transition at which the system resides and efficient coding of information. For the activity transition considered so far, the optimal computational properties are likely to be realized if the information is presented in a rate code, where the activity of a node represents directly an input. To achieve optimal information representation for a synchronization code, where an input is represented by synchronous activity, the system needs to be tuned to the phase transition at the onset of synchronization.

In a system with many parameters, the term *critical point* is misleading. From a mathematical perspective, the critical point is a bifurcation point of the macroscopic dynamics, and as such is characterized by its codimension, which is one in this case (Kutsnetsov, 1998). What this means is that the critical point is actually a manifold which has one dimension less than the embedding parameter space. So, in a one-dimensional parameter space, i.e., when only one parameter is varied, the critical point appears as a (zero-dimensional) point. However, in a two-dimensional parameter space, where a second parameter is varied, we find a (one-dimensional) line of critical points. In a three-dimensional parameter space, criticality occurs on a surface, and so on.

In complex networks, there is an abundance of parameters that affect the dynamics, including for instance the mean degree and mean outgoing link weights, which are often considered, but also clustering coefficients, modularity, and abundances of larger motifs. The precise number of parameters that play a role in neural criticality is hard to determine. However, let us point out that the one dimensional picture (Figure 1), which is usually drawn, is particularly misleading. Consider that in one dimension the probability that two different phase transitions occur at the same parameter value is of measure zero. However, in two parameter dimensions, each phase transition occurs on a critical line in the parameter space, and crossings between the lines are likely. Thus, if there are two processes of plasticity that tune the system to two different critical states, there is generally a possibility to observe both forms of criticality at the same time. Some evidence for such double criticality was already observed by Yang et al. (2012) and Meisel et al. (2013). This can potentially explain why characteristics of both activity (e.g., Beggs and Plenz, 2003, 2004) and synchronization (e.g., Linkenkaer-Hansen et al., 2001; Kitzbichler et al., 2009) phase transitions have been observed in experiments.

So far we have talked about *the brain* as a critical system. However, there is at least the possibility that different regions of the brain are tuned to criticality separately, and perhaps to different phase transitions. Working at the activity transition seems particularly advantageous for the detection of weak stimuli, as it allows a single spike to trigger a cascade of activity. On the other hand, working at the synchronization phase transition appears advantageous for cognitive processes.

## 5. Experimental evidence for the criticality hypothesis

The demonstration of self-organized criticality in the brain is controversial. Several experimental studies support the criticality hypothesis, others interpret their results in contradiction. In this section, we discuss common measurements used to support criticality in the brain and stress their potential shortcomings.

The best proof of criticality would be provided by a phase diagram as in Figure 1, where the critical point appears as a kink in the curve. However, in self-organized critical systems, the control parameter is set by the dynamics itself. If the control parameter is deviated experimentally, it starts to return to its critical value, such that it cannot be set freely. However, if the return is sufficiently slow, phase diagrams can be obtained approximately by monitoring a suitable order parameter while the system relaxes to the critical state.

In recent studies, most evidence for the criticality hypothesis in experiments and simulations is based on power-laws. As power-laws are expected in virtually every critical system, the existence of power-laws is a fundamental prerequisite for criticality, but as such not sufficient to prove criticality. Power-laws have been explained alternatively by different non-critical mechanisms (Touboul and Destexhe, 2010; Marković and Gros, 2014), such as filtered neural activity (Bédard et al., 2006; Bédard and Destexhe, 2009), noise (Bonachela and Muñoz, 2009; Miller et al., 2009), or noisy feed-forward structures amplifying small perturbations (Benayoun et al., 2010). For an educational review on the topic see Beggs and Timme (2012).

**Figure 3:**
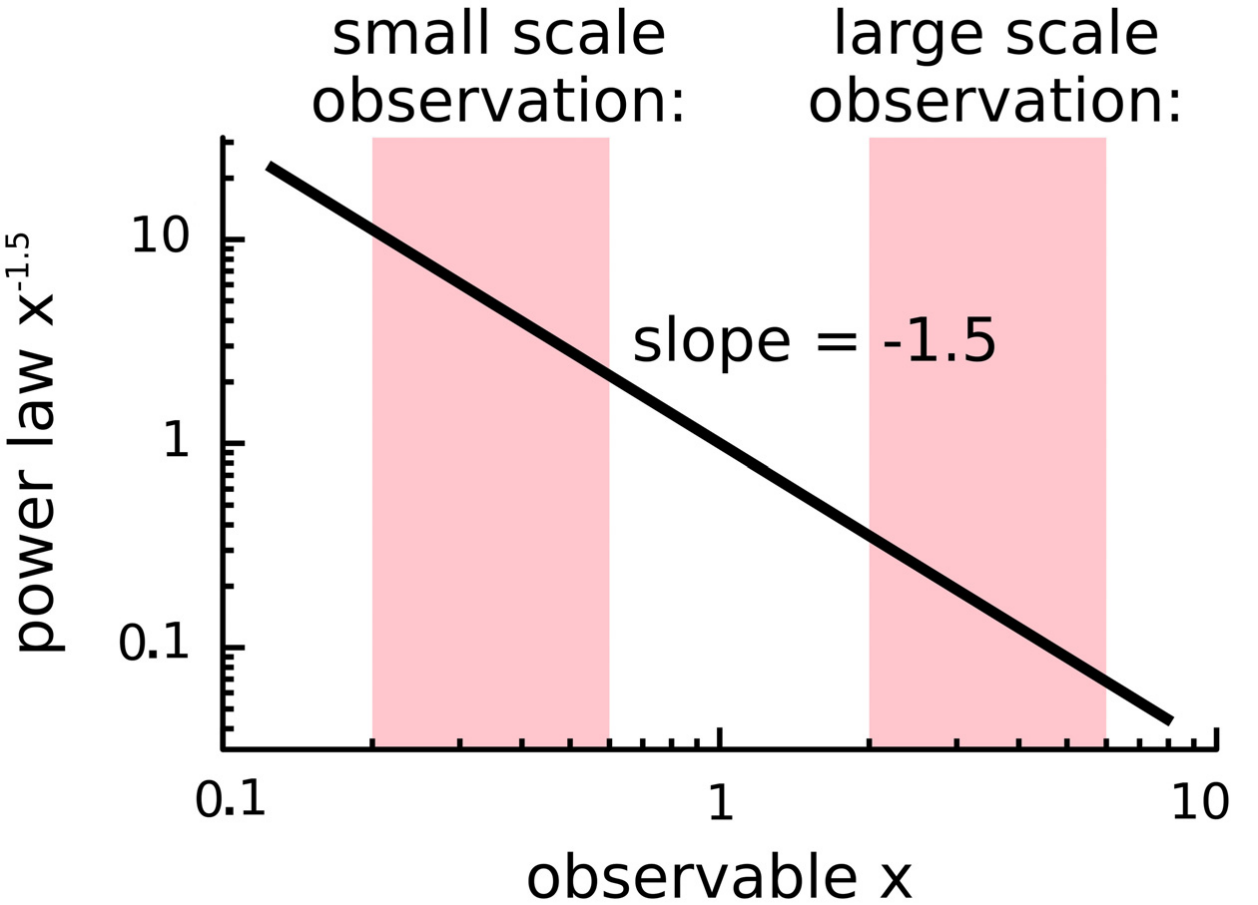
**Scale independence of power-laws**. Plotted is the power-law *f(x)* = *x*^α^ with α = −1.5 on a log-log plot, where *x* is some observable of the system. Independent of the range or scale over which the distribution is measured, power-laws with the same critical exponent are observed.

A major concern is the inference of power-law behavior from data. When plotted on a log-log plot (Figure 3), power-laws follow a straight line with a slope equal to their critical exponent α. However, visual inspection of a diagram can lead to false positives and it has been pointed out that conventional goodness-of-fit tests are ill suited for power-laws (Newman, 2005). The identification of power-laws is thus delicate and demands for advanced fitting procedures because power-laws are difficult to differentiate from other heavy-tail distributions (Clauset et al., 2009; Klaus et al., 2011; Marković and Gros, 2014). Furthermore, power-laws are truncated in systems of finite size (Bonachela et al., 2010) and are influenced by subsampling (Priesemann et al., 2009; Ribeiro et al., 2010; Priesemann et al., 2014; Ribeiro et al., 2014).

Most experimental and numerical studies on self-organized criticality concentrate on the identification of *neuronal avalanches* (Beggs and Plenz, 2003), i.e., bursts of activity that spread through the network and are predicted to follow power-law distributions in certain critical states (e.g., Harris, 1963; Eurich et al., 2002; Larremore et al., 2012). As the precise network topology is often not known in experimental observations, events are considered as part of the same avalanche if they occur in temporal and spatial proximity. This is justified in systems without long-range connections. In this case avalanches form local wave-like structures. If long-range connections are present it is difficult to assign observed activity to a particular avalanche. Avalanches with power-law size distribution can then still be present in the system although no local outbreaks that follow a power-law distribution are detected, which may explain why wave-like activity propagation is for example not observed in acute slices (Stewart and Plenz, 2006).

**Figure 4:**
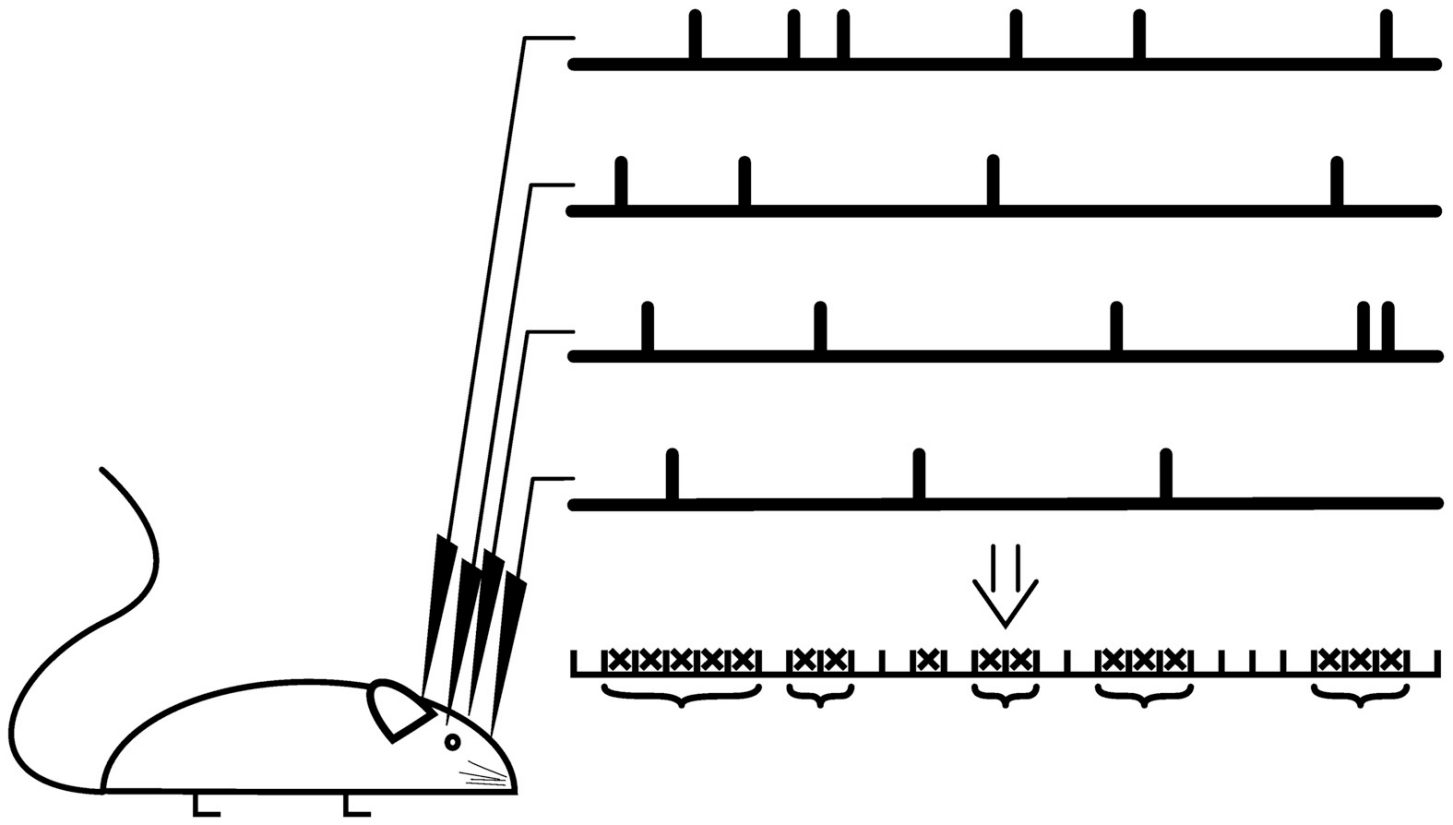
**Avalanche analysis**. Recordings are scanned for specific events such as a negative deflection of the local field potential, which results in a time series for every recording electrode. The event trains are binned and a bin is declared as active (cross) if an event was registered at least at one recording electrode. A suite of active bins is considered as neuronal avalanche (bracket), whose size corresponds to the number of events. For critical neuronal avalanches, the size distribution follows a power-law with a critical exponent close to −1.5.

If the spontaneous activity and the stimulation rate are low, which is the case in most models, one avalanche is temporally separated from the next. In this case, the size of the avalanche is defined as the number of neurons activated by the initial stimulation. In experiments, the definition is not straight-forward as the time-scale separation between dynamics initiation and dynamics progression is less clear (Shew et al., 2009; Ribeiro et al., 2010; Priesemann et al., 2014). Instead, avalanches are declared separated if the dynamics is interrupted for at least one pre-defined time bin. The dynamics is evaluated based on specific events seen in multi-electrode recordings, for example spikes or strong negative deflections of the local field potential (LFP). Resulting event time series are binned and a sequence of consecutive active bins is defined as avalanche, see Figure 4.

In models, in which a time-scale separation between dynamics initiation and dynamics progression is given, the avalanche size distribution is independent of the chosen threshold and bin size (Priesemann et al., 2014), which is a consequence of the scale independence of critical processes. In experimental data, however, avalanche size distributions depend on the chosen event threshold and on the bin size used for the binning process (Pasquale et al., 2008; Touboul and Destexhe, 2010; Priesemann et al., 2013). The avalanche size distribution changes with the bin size when subsampling introduces artificial pauses in single avalanches and when the external input is large enough to initiate multiple avalanches simultaneously (Priesemann et al., 2014). Most studies use a bin size that fits the time that the neural signal takes to spread between electrodes (Beggs and Plenz, 2003; Stewart and Plenz, 2006; Pasquale et al., 2008), some studies also report power-law fitting for different bin sizes (Hahn et al., 2010; Tetzlaff et al., 2010). As expected at criticality, neuronal avalanches show further scale-free properties. Importantly, the avalanche distributions overlap when rescaled by the number of recording electrodes (finite-size scaling, Klaus et al., 2011; Yu et al., 2013). Results are furthermore independent of the recording electrode number and distance (Beggs and Plenz, 2003; Hsu et al., 2008; Pasquale et al., 2008; Tetzlaff et al., 2010) and long range spatial and temporal correlations can be shown (Petermann et al., 2009; Hahn et al., 2010; Yu et al., 2013).

For LFP-recordings, critical neuronal avalanche distributions are reported for various animals and brain regions, both *in vitro* (Beggs and Plenz, 2003, 2004; Mazzoni et al., 2007; Pasquale et al., 2008) and *in vivo* (Petermann et al., 2009; Hahn et al., 2010). Neuronal avalanches can be formed by nested oscillations (slices and anesthetized rat Gireesh and Plenz, 2008) and the variability in the synchronization is maximal (Yang et al., 2012). The critical exponents of the avalanche size distributions (e.g., Beggs and Plenz, 2003; Hahn et al., 2010; Klaus et al., 2011; Friedman et al., 2012) fit theoretical predictions (e.g., Harris, 1963).

When spikes are evaluated, the picture is less consistent. Power-law distributed avalanches were not observed in awake animals (Bédard et al., 2006; Dehghani et al., 2012; Priesemann et al., 2014), which is consistent with theoretical models which predict criticality in a resting state. Indeed there is some evidence that the brain's critical state deteriorates during wakefulness and recovers during sleep (Meisel et al., 2013, compare also Priesemann et al., 2013). In anesthetized animals or cultures, power-law distributions for the spiking activity can be observed (Hahn et al., 2010; Ribeiro et al., 2010), but most recordings do not support power-law fitting (Bédard et al., 2006; Hahn et al., 2010; Ribeiro et al., 2010; Dehghani et al., 2012). Avalanche distributions as observed for spiking activity can however be reproduced by subsampling models implementing self-organized criticality with increased external input and tuned to a slightly subcritical regime (Priesemann et al., 2014). An alternative explanation for non-critical avalanche distributions may be recordings that are biased toward a specific subset of neurons, for example if cell types with particularly clear spike shapes in the extracellular signal are preferentially identified. Furthermore, it is questionable whether we can expect hallmarks of criticality if just a few neurons are recorded simultaneously, because criticality is intrinsically a network effect. In many real-world systems, the scale-independence breaks down if we get too close to the level of single dynamical units.

Apart from properties of the critical state, implications of the self-organization to criticality can be examined. For instance, models of self-organized criticality reproduce developmental phases of cell cultures. Starting from an unconnected state, the temporal development of avalanche distributions in neuronal cultures can be fitted by models of self-organized criticality (Tetzlaff et al., 2010). Also slices from newborn rats of different ages show a temporal development from subcritical to critical dynamics (Gireesh and Plenz, 2008; Stewart and Plenz, 2008). Organotypic cell cultures can develop to subcritical, critical or supercritical states (Pasquale et al., 2008; Tetzlaff et al., 2010). Intriguingly, only the critical cultures show scaling of the mean temporal profile of avalanches, i.e., the data collapse when normalized appropriately (Friedman et al., 2012). The scaling also predicts the relationship between exponents, which is a strong indicator of criticality (Friedman et al., 2012).

Recent results suggest that also in humans, brain dynamics is close to criticality, yet slightly subcritical (Priesemann et al., 2013, 2014), a possibility first raised by Pearlmutter and Houghton (2009). Resting state dynamics from human brains reveal events analogous to neuronal avalanches whose dynamics fluctuate closely around criticality (EEG Allegrini et al., 2010, fMRI Tagliazucchi et al., 2012, MEG Shriki et al., 2013, EEG and MEG during rest and tasks Palva et al., 2013). The resulting critical exponents correlate with the critical exponents of the long-range temporal correlations (Palva et al., 2013). Imaging data suggests furthermore power-law noise because activity fluctuations (e.g., EEG Novikov et al., 1997, ECoG Miller et al., 2009) and correlation fluctuations (e.g., EEG and MEG Linkenkaer-Hansen et al., 2001, fMRI and MEG Kitzbichler et al., 2009) follow power-laws.

Correlations can also be used to construct functional connectivity maps, whose power-law distributed properties might relate to self-organized criticality (e.g., Eguiluz et al., 2005; Bassett et al., 2006; Expert et al., 2010; Lee et al., 2010; Van De Ville et al., 2010). For example, the duration distribution of functional connections in EEG recordings follow power-laws, which are stable over several states of consciousness (awake, loss of consciousness due to anesthesia, and recovery) and frequency bands (Lee et al., 2010).

The criticality hypothesis predicts that sufficiently strong perturbations of the network dynamics should eliminate the power-laws found in the previously cited studies. In the following, we discuss studies showing that observed hallmarks of criticality vanish in response to interventions that change the network dynamics. Such deviations from criticality, and especially the subsequent return of the network to a critical state, strongly support criticality, since alternative explanations of power-laws based on low level features, such as noise and filtering of neuronal tissue, should be independent of the network dynamics.

Hallmarks of criticality are apparently destroyed during epileptic seizures. Epileptic dynamics shows hallmarks of supercritical states, and destroys power-laws observed in healthy brains (Hobbs et al., 2010; Meisel et al., 2012). If the network adapts to the supercritical state during the seizure, this may explain reduced activity and a smaller critical exponent after the seizure (Hsu et al., 2008). A self-organized criticality model suggests a relation between epileptic activity and decreased neuronal connectivity (Meisel et al., 2012). While it is thus tempting to equate epileptic seizures with supercritical dynamics, care has to be taken as seizures could very well be the result of another, overriding mechanism that is not captured by current models of neural self-organized criticality.

In contrast to epileptic seizures, pharmacologically induced variations in activity do not always destroy power-law distributed neuronal avalanches. In acute slices, the level of dopamine that implies maximal activity coincides with critical avalanche size distributions with a critical exponent of −1.5, while more or less dopamine preserves the power-law distribution, but shows steeper critical exponents (Stewart and Plenz, 2006). Steeper exponents reduce the occurrence of large avalanches and spatial correlations (Stewart and Plenz, 2006). Steeper critical exponents are as well observed under reduced spontaneous activity due to pharmacological interventions with a dopamine D1 receptor antagonist (Gireesh and Plenz, 2008), but the same antagonist can also suppress neuronal avalanches (Stewart and Plenz, 2006). The application of acetylcholine, which increases the spontaneous activity, results in exponential avalanche distributions (Pasquale et al., 2008). Strong pharmacological interventions can furthermore change the dynamical state of neural networks via alterations of excitation or inhibition. As expected from the idea that balanced excitation and inhibition are required for critical brain dynamics, this eliminates the observed hallmarks of criticality (Table 1).

**Table 1:** **Deviations from criticality due to unbalanced excitation and inhibition**.

**Alteration of**	**Effect**	**Network state**	**Study**
GABA_A_ receptors	Inhibition ↘	Supercritical	Bimodal avalanche size distributions (cell cultures, Beggs and Plenz, 2003; Mazzoni et al., 2007; Gireesh and Plenz, 2008; Pasquale et al., 2008; Shew et al., 2009, 2011; Yang et al., 2012, intact leech ganglia, Mazzoni et al., 2007, and anesthetized rats, Osorio et al., 2010), with enhanced synchronization (Pasquale et al., 2008; Yang et al., 2012), in particular β-oscillations (Gireesh and Plenz, 2008; Yang et al., 2012), and enhanced correlation (Mazzoni et al., 2007)
Number of inhibitory neurons	Inhibition ↗	Subcritical	Chen et al. (2010)
NMDA receptors (and AMPA in Shew et al., 2009, 2011 and Yang et al., 2012)	Excitation ↘	Subcritical	Exponential avalanche size distributions (Mazzoni et al., 2007; Gireesh and Plenz, 2008; Shew et al., 2009, 2011; Yang et al., 2012) with decreased long-range correlations and large bursts (Mazzoni et al., 2007)

The observation of variable exponents is interesting, as the critical exponents of phase transitions are usually independent of system features. This universality typically holds broadly even across different systems. The exponent of −1.5 is a plausible result as it is characteristic of the directed percolation universality class into which many processes of activity propagation fall. Exponents with a larger absolute value are more difficult to explain. While a complex real world system can potentially exhibit such exponents, it is also plausible that what is observed here is actually the breakdown of the power-law as the system is pushed from the critical state. If this occurs, the underlying distributions return to exponential behavior and thus exhibit less large events. In a certain transition region around the critical state, they can therefore easily be mistaken for steeper power-laws.

The premise of self-organized criticality is that the system is able to tune itself back to the critical state after moderate perturbations. This reorganization to criticality after long-lasting increases in inhibition has so far not been observed experimentally (Tetzlaff et al., 2010). Over the duration of the experiment, the network state does not adapt to decreased inhibition (Shew et al., 2009). Even after the inhibition-decreasing drug is washed out, neuronal slices take several hours to recover criticality (Shew et al., 2009). This time-scale is consistent with reorganization on a slow time-scale, for instance due to slow plasticity mechanisms such as homeostatic plasticity.

In summary, evidence for self-organized criticality is provided by critical neuronal avalanches in various animals, power-law noise in brain imaging data, scale independence and finite-size scaling. While power-laws can also be explained by alternative hypothesis, deviations from criticality and subsequent reorganization provide strong evidence for the criticality hypothesis. Perhaps the most compelling evidence is not provided by any individual study, but rather by the breadth of experimental results which provide evidence for criticality in many different systems using various approaches.

## 6. Models of self-organized criticality in neural networks

Apart from direct experimental evidence, support of self-organized neural criticality comes from a range of models which show that self-organized criticality in the brain is plausible.

While simple model networks allow for analytical considerations that show general features, the more complex models convince with biological detail. Self-organized criticality can be implemented robustly in networks ranging from simple, binary units (e.g., Bienenstock and Lehmann, 1998; Bornholdt and Rohlf, 2000; Bornholdt and Röhl, 2003) up to more biologically realistic integrate-and-fire neurons (e.g., De Arcangelis et al., 2006; Levina et al., 2007, 2009; Meisel and Gross, 2009; Rubinov et al., 2011), for which dynamical switching between subcritical *down*-states and critical *up*-states can be observed (Millman et al., 2010).

Several models reproduced experimental results on criticality (e.g., De Arcangelis et al., 2006; Millman et al., 2010; Tetzlaff et al., 2010; Meisel et al., 2012). Yet, if critical models are suggested by parameter fitting based on experimental data, care has to be taken because the estimation of model parameters shows an intrinsic trend to apparently critical values because, around the phase transition, the uncertainty of the estimate is minimized and the amount of distinguishable models is greatest (Mastromatteo and Marsili, 2011).

Most numerical studies simulate a network of identical model neurons, where activity is regulated by the implemented adaptation mechanism. The network dynamics is launched by an initial stimulation of an arbitrary subset of neuron and analyzed after a period that allows the network to self-organize. The adaptation changes microscopic parameters depending on a given microscopic rule and depending on local measurements of the dynamical state. Using plausible rules, it is then observed that one order parameter of the system approaches the critical point.

If models use activity dependent rules, then the system can self-organize to the critical point at the onset of activity, where avalanche distributions follow power-laws. Inspired by the study of branching processes, these mechanisms change the probability with which activity is transmitted from one neuron to the next. This can be realized either through a regulation of the synaptic connection such as activity-dependent rewiring (Bornholdt and Röhl, 2003; Tetzlaff et al., 2010), Hebbian (De Arcangelis et al., 2006), short-term synaptic plasticity (Levina et al., 2007, 2009; Millman et al., 2010), or, in a certain parameter range, spike-timing dependent plasticity (STDP) (Rubinov et al., 2011); or through a regulation of the neuronal excitability such as internal homeostatic plasticity (Droste et al., 2013).

If the adaption rule is dependent on relative timing or phase differences, the system can self-organize to the critical point at the onset of synchronization. Using phase coherence as order parameter, such models self-organize to criticality if the connections are created and retracted as observed for synaptic rewiring during development, or if the strength of the connections are changed as observed for STDP (Meisel and Gross, 2009). STDP is thus a plausible mechanism that could organize a system to both activity and synchronization phase transitions.

Especially with mechanisms based on STDP, the models reach biologically plausible network structures. They self-organize from a highly connected state to a sparsely connected state, in which only few strong synapses survive (Jost and Kolwankar, 2009; Meisel and Gross, 2009). The resulting networks show power-law distributed synaptic fluctuations (Shin and Kim, 2006) and a scale-free network structure (Shin and Kim, 2006; Meisel and Gross, 2009).

Most neuron models are rather simple, but the self-organized criticality mechanisms also allow for the implementation of certain more realistic properties. Models using intergrate-and-fire neurons can implement delayed synaptic transmission (e.g., Rubinov et al., 2011) and a refractory period, which is thought to hinder back-propagation of neuronal avalanches (e.g., De Arcangelis et al., 2006). In addition, integrate-and-fire neurons can also have leaky membranes (Meisel and Gross, 2009; Millman et al., 2010; Rubinov et al., 2011). Up to now, self-organized criticality has not been reported for conductance-based neuron models, probably because the network simulations are constraint by the available computational power; limiting the self-organization to criticality by restricting either network size or simulation duration. Just one study reports that a network of Hodgkin-Huxley model neurons self-organizes to a scale-free network with STDP (Shin and Kim, 2006). The observation of self-organized criticality across a wide range of neuron models is intuitive as the critical state itself should be independent of microscopic details.

Criticality and self-organized criticality can already be observed in models with very simple dynamics as the toy model proposed above. Nevertheless, many current models capture the complex interplay between inhibitory and excitatory neurons (De Arcangelis et al., 2006; Shin and Kim, 2006; Tetzlaff et al., 2010). The resulting dynamics then depends only on the ratio of inhibitory and excitatory connection strengths such that a regulation of the excitatory connections is sufficient (Bienenstock and Lehmann, 1998; Shin and Kim, 2006). The exact role played by the balance of excitation and inhibition in the brain is poorly understood. It can be shown mathematically that this interplay in itself is not a prerequisite for criticality (Jost and Kolwankar, 2009). Nevertheless, the interplay between inhibition and excitation could play an important role for the system's computational capabilities in the critical state.

A crucial ingredient for robust self-organized criticality is the ability to sense the global state of the system based on local information. For instance, concerning the activity transition, every local neuron or synapse has a plasticity rule that increases or decreases the unit's activity. Self-organized criticality can only be achieved if the increase is more frequently or more strongly realized in the subcritical than in the supercritical state, and the decrease in the supercritical state. Thus, on some level, the global state has to be detectable on the local scale. Regarding activity, this is much easier for the supercritical state than for the subcritical state. Even a single neuron or synapse that experiences a high level of activity can conclude that the system is in the supercritical state with high probability. Conversely, the absence of such activity, observed locally, does not necessitate a subcritical state on the global level.

Because the subcritical state is difficult to recognize by a local mechanism, it is likely that criticality in the brain is achieved by a slow continuous increase of the control parameter, which is then overcompensated by a decisive decrease once supercritical dynamics is detected. Such an asymmetric regulation is implemented in models inspired by short term synaptic depression, where synaptic efficiency is abruptly decrease when a spike occurs, and afterwards exponentially increased until the next spike occurs (Levina et al., 2007, 2009; Millman et al., 2010), and in a model inspired by calcium dependent development of axons and dendrites with faster dynamics in the direction of subcritical states, where the rate of the dendritic retraction was twice the rate of the axonal outgrowth (Tetzlaff et al., 2010). The use of asymmetric regulation was emphasized in a simpler model by Droste et al. (2013). Since the dynamics on the subcritical side is slower, the system spends more time on the subcritical side and thus, in average, appears slightly subcritical, which is consistent with experimental findings.

In general, we can expect that self-organized criticality in finite systems drives the system slightly into the subcritical phase. For the onset of synchronization, the local detection of synchrony implies that some degree of synchrony exists in the system such that the system must be in the supercritical state. By contrast, the absence of synchrony observed locally does not imply that the system is necessarily in the subcritical state as synchronous dynamics may already exist elsewhere in the system. Again, we expect that self-organization will drive the system to a slightly subcritical state.

The finding that already highly simplified models reproduce experimental results suggests fundamental properties of self-organizing mechanisms for which implementation details do not matter. The robustness of self-organization to criticality can increase with system size, suggesting that self-organized criticality is especially easily implemented in large neural networks (Levina et al., 2007; Rubinov et al., 2011). While each of the models discussed here can be criticized in various ways, the observation of robust self-organized criticality across a broad range of modeling assumptions and frameworks lends much credibility to the criticality hypothesis.

## 7. Discussion

If self-organized criticality is indeed fundamental for the functioning of the brain, then we expect a link between self-organized criticality and other properties of the brain. In the following, we speculate on the relation of self-organized criticality with sensory input, learning and sleep.

Most studies of self-organized criticality have so far focused on systems without input. However, to assess the impact of criticality on the brain's computational capabilities, inputs need to be considered. Based on current results, it is likely that high levels of input will cause hallmarks of criticality to disappear as internal dynamics is replaced by externally triggered activity. Inputs are considerably decreased in slice and cell cultures compared to *in vivo* preparations, and the same probably holds for anesthetized animals compared to awake animals (Beggs and Plenz, 2003; Ribeiro et al., 2010; Touboul and Destexhe, 2010). It is therefore not surprising that most evidence for criticality comes from these systems. Future experimental studies aiming to find hallmarks of criticality should therefore likewise focus on low-input situations.

In systems with strong input, the discussion of self-organized criticality is conceptually more difficult as the definition of the system now has to include a statistical model of inputs. While it is still possible to define phases and phase transitions, the phase transitions become harder to identify and critical states can easily be mistaken for supercritical states. For instance, if we add inputs to the toy model proposed above we always observe activity, even in subcritical states.

In a situation where the brain is exposed to a significant level of input, we would expect that self-tuning mechanisms fail as the retuning mechanisms start to compensate for the input by regulating activity down. The system thus departs from the state where the internally generated dynamics is critical. Indeed, evidence for critical brain dynamics decreases during prolongated periods of wakefulness, and increases after a night of sleep (Meisel et al., 2013). It is thus plausible that sleep is essential for retuning the brain to the critical state where it can operate effectively.

Both experimental studies (e.g., Bassett et al., 2006; Bédard et al., 2006; Hahn et al., 2010; Dehghani et al., 2012; Priesemann et al., 2013, 2014) and models point to self-organization to a subcritical state close to criticality. Many authors have suggested that this is a safety mechanism to prevent pathological supercritical dynamics. From a theoretical point, another explanation appears more plausible. Any finite real world system, subject to noise and inputs, can only self-organize to critical states with given accuracy. Due to limitations in the sensing of the global state, systems spend in average more time in the subcritical phase.

One property that is so far widely ignored in the literature is the dimensionality of the underlying parameter space. In simple systems that have only one control parameter the critical state is a point. However, in general it is a manifold whose dimensionality is less than the dimensionality of the parameter space. Technically, the parameter space spanned by a complex network includes all the individual link weights and is thus almost infinite. Even if we only focus on the main macroscopic descriptors of networks we can easily identify tens of parameters that can potentially affect the dynamics and can be affected by the plasticity. If only ten such parameters played a role in the real system the critical state would still be a nine dimensional manifold and thus a huge parameter space.

One implication of the high dimensionality of the critical manifold is that the system can change and therefore learn while remaining in the critical state. However, the connection between learning and criticality goes apparently deeper than that. For instance it has been claimed that self-organized criticality is essential for learning, for review see Hsu et al. (2008), but further explorations of the detailed connection between learning and criticality seem necessary.

Another implication of the high-dimensional parameter space of complex networks is that the system can reside in multiple phase transitions at the same time. Intriguingly, recent results suggest that neural networks are organized to both the activity and synchronization phase transition (e.g., Yang et al., 2012 for organotypic slices, Meisel et al. (2013), or Linkenkaer-Hansen et al. (2001); Kitzbichler et al. (2009) compared to Tagliazucchi et al. (2012); Shriki et al. (2013) for brain imaging). Future modeling work should address whether neural networks can support multiple or simultaneous critical states.

A central question is whether the brain self-organizes to criticality as a single system, or as a collection of many, potentially overlapping, subsystems. While simulations consider predominantly homogeneous networks, anatomical features divide the brain in clearly defined brain areas. Several authors stress the possibility that different brain areas self-organize independently (Bédard et al., 2006; Kitzbichler et al., 2009; Meisel and Gross, 2009; Priesemann et al., 2009; Meisel et al., 2012). If this is confirmed the next logical questions are if all brain areas self-organize to criticality, and if yes, do they all organize to the same phase transition? Resolving these questions could greatly strengthen the link between self-organized criticality and its medical implications.

## 8. Conclusion

The neural criticality hypothesis is motivated by the relationship between criticality and optimal computational properties. The hypothesis is supported by experiments that observed hallmarks of criticality for a wide range of animals from leech to humans, over several states of consciousness, and on many different experimental scales from recordings of few neurons up to the whole brain. However, the experimental evidence is still controversial and more studies are needed to resolve major open questions and rule out alternative explanations for the observed phenomena. Based on the presently available work, we judge self-organized as preferable over alternative explanations because it provides an evolutionarily-motivated explanation for several otherwise disconnected observation.

In addition to experiments, the criticality hypothesis is supported by models which demonstrate that the self-organization to critical states in the brain is feasible and plausible. While these models necessarily simplify the brain to various degrees, they paint a consistent picture where essentially the same phenomenon is observed independently of specific modeling assumptions.

The criticality hypothesis is intriguing because it opens new perspectives in several areas. First, deviations from criticality could be symptomatic of diseases of the central nervous system (Meisel et al., 2012; Shew and Plenz, 2013). Understanding self-organized criticality in the brain could thus lead to new diagnostic tools, and possibly treatments. Second, connections are presently emerging which suggest that understanding criticality in the brain could provide important insights into other phenomena including sleep, learning, the root-causes of certain diseases, and a deeper understanding of information processing. Finally, several results which have been obtained in the context of self-organized criticality in the brain suggest that criticality is a prerequisite for efficient information processing in unstructured systems. This could provide a general principle that is broadly relevant beyond the field of neuroscience and could be valuable for overcoming various challenges, from understanding swarm intelligence (Ioannou et al., 2012) to constructing microprocessors that process information using randomly-deposited nano-scale components. We believe that these perspectives provide a strong incentive for more experimental and theoretical work in the area of self-organized criticality.

## Author contributions

Janina Hesse scanned the literature and wrote the paper, Thilo Gross wrote the paper and supervised the project.

## Funding

This work was partially supported by the EPSRC under grant no EP/K031686/1. Janina Hesse was funded by a scholarship from the ÉEcole Normale Sup#x000E9;rieure, Paris, and by grants from the Federal Ministry of Education and Research, Germany (01GQ1001A, 01GQ0901) and the Deutsche Forschungsgemeinschaft (SFB618, GK1589/1).

### Conflict of interest statement

The authors declare that the research was conducted in the absence of any commercial or financial relationships that could be construed as a potential conflict of interest.

## References

[B1] Allegrini P. Paradisi P. Menicucci D. Gemignani A. (2010). Fractal complexity in spontaneous EEG metastable-state transitions: new vistas on integrated neural dynamics. Front. Physiol. 1:128 10.3389/fphys.2010.00128 21423370 PMC3059954

[B2] Bak P. (1996). How Nature Works. New York, NY: Copernicus 10.1007/978-1-4757-5426-1

[B3] Bak P. Tang C. Wiesenfeld K. (1988). Self-organized criticality. Phys. Rev. A 38, 364–374 10.1103/PhysRevA.38.364 9900174

[B4] Barabási A.-L. Albert R. (1999). Emergence of scaling in random networks. Science 286, 509–512 10.1126/science.286.5439.509 10521342

[B5] Bassett D. Meyer-Lindenberg A. Achard S. Duke T. Bullmore E. (2006). Adaptive reconfiguration of fractal small-world human brain functional networks. Proc. Natl. Acad. Sci. U.S.A. 103:19518 10.1073/pnas.0606005103 17159150 PMC1838565

[B6] Bédard C. Destexhe A. (2009). Macroscopic models of local field potentials and the apparent 1/f noise in brain activity. Biophys. J. 96, 2589–2603 10.1016/j.bpj.2008.12.3951 19348744 PMC2711281

[B7] Bédard C. Kroeger H. Destexhe A. (2006). Does the 1/f frequency scaling of brain signals reflect self-organized critical states? Phys. Rev. Lett. 97:118102 10.1103/PhysRevLett.97.118102 17025932

[B8] Beggs J. (2008). The criticality hypothesis: how local cortical networks might optimize information processing. Philos. Trans. R. Soc. A Math. Phys. Eng. Sci. 366:329 10.1098/rsta.2007.2092 17673410

[B9] Beggs J. Plenz D. (2003). Neuronal avalanches in neocortical circuits. J. Neurosci. 23:11167 14657176 10.1523/JNEUROSCI.23-35-11167.2003 PMC6741045

[B10] Beggs J. Plenz D. (2004). Neuronal avalanches are diverse and precise activity patterns that are stable for many hours in cortical slice cultures. J. Neurosci. 24:5216 10.1523/JNEUROSCI.0540-04.2004 15175392 PMC6729198

[B11] Beggs J. M. Timme N. (2012). Being critical of criticality in the brain. Front. Physiol. 3:163 10.3389/fphys.2012.00163 22701101 PMC3369250

[B12] Benayoun M. Cowan J. Van Drongelen W. Wallace E. (2010). Avalanches in a stochastic model of spiking neurons. PLoS Comput. Biol. 6:e1000846 10.1371/journal.pcbi.1000846 20628615 PMC2900286

[B13] Bertschinger N. Natschläger T. (2004). Real-time computation at the edge of chaos in recurrent neural networks. Neural Comput. 16, 1413–1436 10.1162/089976604323057443 15165396

[B14] Bienenstock E. Lehmann D. (1998). Regulated criticality in the brain? Adv. Complex Syst. 1, 361–384 10.1142/S0219525998000223

[B15] Bonachela J. De Franciscis S. Torres J. Muñoz M. (2010). Self-organization without conservation: are neuronal avalanches generically critical? J. Stat. Mech. Theory Exp. 2010:P02015 10.1088/1742-5468/2010/02/P02015

[B16] Bonachela J. Muñoz M. (2009). Self-organization without conservation: true or just apparent scale-invariance? J. Stat. Mech. Theory Exp. 2009:P09009 10.1088/1742-5468/2009/09/P0900923030099

[B17] Bornholdt S. Röhl T. (2003). Self-organized critical neural networks. Phys. Rev. E 67:066118 10.1103/PhysRevE.67.066118 16241315

[B18] Bornholdt S. Rohlf T. (2000). Topological evolution of dynamical networks: Global criticality from local dynamics. Phys. Rev. Lett. 84, 6114–6117 10.1103/PhysRevLett.84.6114 10991137

[B19] Botcharova M. Farmer S. F. Berthouze L. (2012). A power-law distribution of phase-locking intervals does not imply critical interaction. Phys. Rev. E Stat. Nonlin. Soft Matter Phys. 86(5 Pt 1):051920 10.1103/PhysRevE.86.051920 23214827

[B20] Cencini M. Falcioni M. Olbrich E. Kantz H. Vulpiani A. (2000). Chaos or noise: difficulties of a distinction. Phys. Rev. E 62:427 10.1103/PhysRevE.62.427 11088477

[B21] Chen W. Hobbs J. Tang A. Beggs J. (2010). A few strong connections: optimizing information retention in neuronal avalanches. BMC Neurosci. 11:3 10.1186/1471-2202-11-3 20053290 PMC2824798

[B22] Chialvo D. (2006). Are our senses critical. Nat. Phys. 2, 301–302 10.1038/nphys300

[B23] Clauset A. Shalizi C. R. Newman M. E. J. (2009). Power-law distributions in empirical data. SIAM Rev. 51, 661–703 10.1137/070710111

[B24] De Arcangelis L. Perrone-Capano C. Herrmann H. (2006). Self-organized criticality model for brain plasticity. Phys. Rev. Lett. 96:28107 10.1103/PhysRevLett.96.028107 16486652

[B25] Dehghani N. Hatsopoulos N. G. Haga Z. D. Parker R. A. Greger B. Halgren E. (2012). Avalanche analysis from multielectrode ensemble recordings in cat, monkey, and human cerebral cortex during wakefulness and sleep. Front. Physiol. 3:302 10.3389/fphys.2012.00302 22934053 PMC3429073

[B26] Derrida B. Pomeau Y. (1986). Random networks of automata: a simple annealed approximation. Euro. Lett. 1:45 10.1209/0295-5075/1/2/001

[B27] Dickman R. Muñoz M. Vespignani A. Zapperi S. (2000). Paths to self-organized criticality. Brazil. J. Phys. 30, 27–41 10.1590/S0103-97332000000100004

[B28] Droste F. Do A.-L. Gross T. (2013). Analytical investigation of self-organized criticality in neural networks. J. R. Soc. Interface 10:20120558 10.1098/rsif.2012.0558 22977096 PMC3565782

[B29] Eguiluz V. Chialvo D. Cecchi G. Baliki M. Apkarian A. (2005). Scale-free brain functional networks. Phys. Rev. Lett. 94:18102 10.1103/PhysRevLett.94.018102 15698136

[B30] Eurich C. Herrmann J. Ernst U. (2002). Finite-size effects of avalanche dynamics. Phys. Rev. E 66:066137 10.1103/PhysRevE.66.066137 12513377

[B31] Expert P. Lambiotte R. Chialvo D. R. Christensen K. Jensen H. J. Sharp D. J. (2010). Self-similar correlation function in brain resting-state functional magnetic resonance imaging. J. R. Soc. Interface 8, 472–479 10.1098/rsif.2010.0416 20861038 PMC3061122

[B32] Frette V. Christensen K. Malthe-Sørenssen A. Feder J. Jøssang T. Meakin P. (1996). Avalanche dynamics in a pile of rice. Nature 379, 49–52 10.1038/379049a0

[B33] Friedman N. Ito S. Brinkman B. A. Shimono M. DeVille R. L. Dahmen K. A. (2012). Universal critical dynamics in high resolution neuronal avalanche data. Phys. Rev. Lett. 108:208102 10.1103/PhysRevLett.108.208102 23003192

[B34] Gireesh E. Plenz D. (2008). Neuronal avalanches organize as nested theta-and beta/gamma-oscillations during development of cortical layer 2/3. Proc. Natl. Acad. Sci. U.S.A. 105:7576 10.1073/pnas.0800537105 18499802 PMC2396689

[B35] Goldenfeld N. (1992). Lectures on Phase Transitions and the Renormalization Group. Reading: Addison-Wesley, Advanced Book Program

[B36] Guckenheimer J. Holmes P. (1983). Nonlinear Oscillations, Dynamical Systems, and Bifurcations of Vector Fields, Vol. 42 New York, NY: Springer Verlag 10.1007/978-1-4612-1140-2

[B37] Gutiérrez R. Amann A. Assenza S. Gómez-Gardenes J. Latora V. Boccaletti S. (2011). Emerging meso-and macroscales from synchronization of adaptive networks. Phys. Rev. Lett. 107:234103 10.1103/PhysRevLett.107.234103 22182093

[B38] Hahn G. Petermann T. Havenith M. Yu S. Singer W. Plenz D. (2010). Neuronal avalanches in spontaneous activity *in vivo*. J. Neurophysiol. 104:3312 10.1152/jn.00953.2009 20631221 PMC3007625

[B39] Haldeman C. Beggs J. (2005). Critical branching captures activity in living neural networks and maximizes the number of metastable states. Phys. Rev. Lett. 94:58101 10.1103/PhysRevLett.94.058101 15783702

[B40] Harris T. E. (1963). The Theory of Branching Processes. Berlin: Springer Verlag, 232

[B41] Hausdorff J. M. Peng C.-K. (1996). Multiscaled randomness: a possible source of 1/f noise in biology. Phys. Rev. E 54:2154 9965304 10.1103/physreve.54.2154

[B42] Hobbs J. Smith J. Beggs J. (2010). Aberrant neuronal avalanches in cortical tissue removed from juvenile epilepsy patients. J. Clin. Neurophysiol. 27:380 10.1097/WNP.0b013e3181fdf8d3 21076327

[B43] Hsu D. Chen W. Hsu M. Beggs J. (2008). An open hypothesis: is epilepsy learned, and can it be unlearned? Epilep. Behav. 13, 511–522 10.1016/j.yebeh.2008.05.007 18573694 PMC2611958

[B44] Ioannou C. Guttal V. Couzin I. (2012). Predatory fish select for coordinated collective motion in virtual prey. Science 337, 1212–1215 10.1126/science.1218919 22903520

[B45] Jost J. Kolwankar K. (2009). Evolution of network structure by temporal learning. Phys. A Stat. Mech. Appl. 388, 1959–1966 10.1016/j.physa.2008.12.073

[B46] Kauffman S. (1984). Emergent properties in random complex automata. Phys. D Nonlin. Phenom. 10, 145–156 10.1016/0167-2789(84)90257-4

[B47] Kello C. Brown G. Ferrer-i Cancho R. Holden J. Linkenkaer-Hansen K. Rhodes T. (2010). Scaling laws in cognitive sciences. Trends Cogn. Sci. 14, 223–232 10.1016/j.tics.2010.02.005 20363176

[B48] Kinouchi O. Copelli M. (2006). Optimal dynamical range of excitable networks at criticality. Nat. Phys. 2, 348–351 10.1038/nphys289

[B49] Kitzbichler M. Smith M. Christensen S. Bullmore E. (2009). Broadband criticality of human brain network synchronization. PLoS Comput. Biol. 5:e1000314 10.1371/journal.pcbi.1000314 19300473 PMC2647739

[B50] Klaus A. Yu S. Plenz D. (2011). Statistical analyses support power law distributions found in neuronal avalanches. PLoS ONE 6:e19779 10.1371/journal.pone.0019779 21720544 PMC3102672

[B51] Krapivsky P. Krioukov D. (2008). Scale-free networks as preasymptotic regimes of superlinear preferential attachment. Phys. Rev. E 78:026114 10.1103/PhysRevE.78.026114 18850904

[B52] Kutsnetsov I. A. (1998). Elements of Applied Bifurcation Theory, Vol. 112 New York, NY: Springer

[B53] Langton C. (1990). Computation at the edge of chaos: phase transitions and emergent computation. Phys. D Nonlin. Phenom. 42, 12–37 10.1016/0167-2789(90)90064-V

[B54] Larremore D. B. Carpenter M. Y. Ott E. Restrepo J. G. (2012). Statistical properties of avalanches in networks. Phys. Rev. E 85:066131 10.1103/PhysRevE.85.066131 23005186

[B55] Lee U. Oh G. Kim S. Noh G. Choi B. Mashour G. (2010). Brain networks maintain a scale-free organization across consciousness, anesthesia, and recovery: evidence for adaptive reconfiguration. Anesthesiology 113:1081 10.1097/ALN.0b013e3181f229b5 20881595 PMC2962769

[B56] Legenstein R. Maass W. (2007). What makes a dynamical system computationally powerful? in New Directions in Statistical Signal Processing: From Systems to Brain, eds Haykin S. Principe J. C. Sejnowski T. McWhirter J. (Cambridge, MIT Press), 127–154

[B57] Levina A. Herrmann J. Geisel T. (2007). Dynamical synapses causing self-organized criticality in neural networks. Nat. Phys. 3, 857–860 10.1038/nphys758

[B58] Levina A. Herrmann J. Geisel T. (2009). Phase transitions towards criticality in a neural system with adaptive interactions. Phys. Rev. Lett. 102:118110 10.1103/PhysRevLett.102.118110 19392248

[B59] Levy M. Solomon S. (1996). Power laws are logarithmic Boltzmann laws. Int. J. Modern Phys. C 7, 595–601 10.1142/S012918319600049123231250

[B60] Linkenkaer-Hansen K. Nikouline V. V. Palva J. M. Ilmoniemi R. J. (2001). Long-range temporal correlations and scaling behavior in human brain oscillations. J. Neurosci. 21, 1370–1377 11160408 10.1523/JNEUROSCI.21-04-01370.2001 PMC6762238

[B61] Maass W. Natschläger T. Markram H. (2002). Real-time computing without stable states: a new framework for neural computation based on perturbations. Neural Comput. 14, 2531–2560 10.1162/089976602760407955 12433288

[B62] Marković D. Gros C. (2014). Power laws and self-organized criticality in theory and nature. Phys. Rep. 536, 41–74 10.1016/j.physrep.2013.11.002

[B63] Mastromatteo I. Marsili M. (2011). On the criticality of inferred models. J. Stat. Mech. Theory Exp. 2011:P10012 10.1088/1742-5468/2011/10/P10012

[B64] Mazzoni A. Broccard F. Garcia-Perez E. Bonifazi P. Ruaro M. Torre V. (2007). On the dynamics of the spontaneous activity in neuronal networks. PLoS ONE 2:e439 10.1371/journal.pone.0000439 17502919 PMC1857824

[B65] Meisel C. Gross T. (2009). Adaptive self-organization in a realistic neural network model. Phys. Rev. E 80:061917 10.1103/PhysRevE.80.061917 20365200

[B66] Meisel C. Olbrich E. Shriki O. Achermann P. (2013). Fading signatures of critical brain dynamics during sustained wakefulness in humans. J. Neurosci. 33, 17363–17372 10.1523/JNEUROSCI.1516-13.2013 24174669 PMC3858643

[B67] Meisel C. Storch A. Hallmeyer-Elgner S. Bullmore E. Gross T. (2012). Failure of adaptive self-organized criticality during epileptic seizure attacks. PLoS Comput. Biol. 8:e1002312 10.1371/journal.pcbi.1002312 22241971 PMC3252275

[B68] Miller K. Sorensen L. Ojemann J. Den Nijs M. (2009). Power-law scaling in the brain surface electric potential. PLoS Comput. Biol. 5:e1000609 10.1371/journal.pcbi.1000609 20019800 PMC2787015

[B69] Millman D. Mihalas S. Kirkwood A. Niebur E. (2010). Self-organized criticality occurs in non-conservative neuronal networks during up-states. Nat. Phys. 6, 801–805 10.1038/nphys1757 21804861 PMC3145974

[B70] Moretti P. Muñoz M. A. (2013). Griffiths phases and the stretching of criticality in brain networks. Nat. Commun. 4:2521 10.1038/ncomms3521 24088740

[B71] Newman M. E. (2005). Power laws, pareto distributions and zipf's law. Contemp. Phys. 46, 323–351 10.1080/00107510500052444

[B72] Novikov E. Novikov A. Shannahoff-Khalsa D. Schwartz B. Wright J. (1997). Scale-similar activity in the brain. Phys. Rev. E 56, 2387–2389 10.1103/PhysRevE.56.R238717554000

[B73] Osorio I. Frei M. Sornette D. Milton J. Lai Y. (2010). Epileptic seizures: quakes of the brain? Phys. Rev. E 82:021919 10.1103/PhysRevE.82.021919 20866849

[B74] Palva J. M. Zhigalov A. Hirvonen J. Korhonen O. Linkenkaer-Hansen K. Palva S. (2013). Neuronal long-range temporal correlations and avalanche dynamics are correlated with behavioral scaling laws. Proc. Natl. Acad. Sci. U.S.A. 110, 3585–3590 10.1073/pnas.1216855110 23401536 PMC3587255

[B75] Pasquale V. Massobrio P. Bologna L. Chiappalone M. Martinoia S. (2008). Self-organization and neuronal avalanches in networks of dissociated cortical neurons. Neuroscience 153, 1354–1369 10.1016/j.neuroscience.2008.03.050 18448256

[B76] Pearlmutter B. Houghton C. (2009). A new hypothesis for sleep: tuning for criticality. Neural Comput. 21, 1622–1641 10.1162/neco.2009.05-08-787 19191602

[B77] Petermann T. Thiagarajan T. Lebedev M. Nicolelis M. Chialvo D. Plenz D. (2009). Spontaneous cortical activity in awake monkeys composed of neuronal avalanches. Proc. Natl. Acad. Sci. U.S.A. 106:15921 10.1073/pnas.0904089106 19717463 PMC2732708

[B78] Plenz D. Niebur E. (2014). Criticality in Neural Systems. Series ed. Schuster H. G. (Weinheim: John Wiley & Sons).

[B79] Priesemann V. Munk M. Wibral M. (2009). Subsampling effects in neuronal avalanche distributions recorded *in vivo*. BMC Neurosci. 10:40 10.1186/1471-2202-10-40 19400967 PMC2697147

[B80] Priesemann V. Valderrama M. Wibral M. Le Van Quyen M. (2013). Neuronal avalanches differ from wakefulness to deep sleep – evidence from intracranial depth recordings in humans. PLoS Comput. Biol. 9:e1002985 10.1371/journal.pcbi.1002985 23555220 PMC3605058

[B81] Priesemann V. Wibral M. Valderrama M. Pröpper R. Le Van Quyen M. Geisel T. (2014). Spike avalanches *in vivo* suggest a driven, slightly subcritical brain state. Front. Syst. Neurosci. 8:108 10.3389/fnsys.2014.00108 25009473 PMC4068003

[B82] Ribeiro T. L. Copelli M. Caixeta F. Belchior H. Chialvo D. R. Nicolelis M. A. (2010). Spike avalanches exhibit universal dynamics across the sleep-wake cycle. PLoS ONE 5:e14129 10.1371/journal.pone.0014129 21152422 PMC2994706

[B83] Ribeiro T. L. Ribeiro S. Belchior H. Caixeta F. Copelli M. (2014). Undersampled critical branching processes on small-world and random networks fail to reproduce the statistics of spike avalanches. PLoS ONE 9:e94992 10.1371/journal.pone.0094992 24751599 PMC3994033

[B84] Rubinov M. Sporns O. Thivierge J.-P. Breakspear M. (2011). Neurobiologically realistic determinants of self-organized criticality in networks of spiking neurons. PLoS Comput. Biol. 7:e1002038 10.1371/journal.pcbi.1002038 21673863 PMC3107249

[B85] Scheffer M. Bascompte J. Brock W. A. Brovkin V. Carpenter S. R. Dakos V. (2009). Early-warning signals for critical transitions. Nature 461, 53–59 10.1038/nature08227 19727193

[B86] Shew W. Yang H. Petermann T. Roy R. Plenz D. (2009). Neuronal avalanches imply maximum dynamic range in cortical networks at criticality. J. Neurosci. 29:15595 10.1523/JNEUROSCI.3864-09.2009 20007483 PMC3862241

[B87] Shew W. L. Plenz D. (2013). The functional benefits of criticality in the cortex. Neuroscientist 19, 88–100 10.1177/1073858412445487 22627091

[B88] Shew W. L. Yang H. Yu S. Roy R. Plenz D. (2011). Information capacity and transmission are maximized in balanced cortical networks with neuronal avalanches. J. Neurosci. 31, 55–63 10.1523/JNEUROSCI.4637-10.2011 21209189 PMC3082868

[B89] Shin C. Kim S. (2006). Self-organized criticality and scale-free properties in emergent functional neural networks. Phys. Rev. E 74:045101 10.1103/PhysRevE.74.045101 17155118

[B90] Shriki O. Alstott J. Carver F. Holroyd T. Henson R. N. Smith M. L. (2013). Neuronal avalanches in the resting MEG of the human brain. J. Neurosci. 33, 7079–7090 10.1523/JNEUROSCI.4286-12.2013 23595765 PMC3665287

[B91] Stewart C. Plenz D. (2006). Inverted-U profile of dopamine – NMDA-mediated spontaneous avalanche recurrence in superficial layers of rat prefrontal cortex. J. Neurosci. 26, 8148–8159 10.1523/JNEUROSCI.0723-06.2006 16885228 PMC6673780

[B92] Stewart C. V. Plenz D. (2008). Homeostasis of neuronal avalanches during postnatal cortex development *in vitro*. J. Neurosci. Methods 169, 405–416 10.1016/j.jneumeth.2007.10.021 18082894 PMC2743406

[B93] Tagliazucchi E. Balenzuela P. Fraiman D. Chialvo D. R. (2012). Criticality in large-scale brain fMRI dynamics unveiled by a novel point process analysis. Front. Physiol. 3:15 10.3389/fphys.2012.00015 22347863 PMC3274757

[B94] Tetzlaff C. Okujeni S. Egert U. Wörgötter F. Butz M. (2010). Self-organized criticality in developing neuronal networks. PLoS Comput. Biol. 6:e1001013 10.1371/journal.pcbi.1001013 21152008 PMC2996321

[B95] Touboul J. Destexhe A. (2010). Can power-law scaling and neuronal avalanches arise from stochastic dynamics. PLoS ONE 5:e8982 10.1371/journal.pone.0008982 20161798 PMC2820096

[B96] Van De Ville D. Britz J. Michel C. (2010). EEG microstate sequences in healthy humans at rest reveal scale-free dynamics. Proc. Natl. Acad. Sci. U.S.A. 107, 18179–18184 10.1073/pnas.1007841107 20921381 PMC2964192

[B97] Vespignani A. Zapperi S. (1998). How self-organized criticality works: a unified mean-field picture. Phys. Rev. E 57:6345 10.1103/PhysRevE.57.6345

[B98] Yang H. Shew W. L. Roy R. Plenz D. (2012). Maximal variability of phase synchrony in cortical networks with neuronal avalanches. J. Neurosci. 32, 1061–1072 10.1523/JNEUROSCI.2771-11.2012 22262904 PMC3319677

[B99] Yu S. Klaus A. Yang H. Plenz D. (2014). Scale-invariant neuronal avalanche dynamics and the cut-off in size distributions. PLoS ONE 9:e99761 10.1371/journal.pone.0099761 24927158 PMC4057403

[B100] Yu S. Yang H. Shriki O. Plenz D. (2013). Universal organization of resting brain activity at the thermodynamic critical point. Front. Syst. Neurosci. 7:42 10.3389/fnsys.2013.00042 23986660 PMC3749752

